# Notch1 Is Not Required for Acinar-to-Ductal Metaplasia in a Model of Kras-Induced Pancreatic Ductal Adenocarcinoma

**DOI:** 10.1371/journal.pone.0052133

**Published:** 2012-12-19

**Authors:** Jacqueline L. Avila, Scott Troutman, Amy Durham, Joseph L. Kissil

**Affiliations:** 1 Molecular and Cellular Oncogenesis Program, The Wistar Institute, Philadelphia, Pennsylvania, United States of America; 2 Department of Pathobiology, University of Pennsylvania School of Veterinary Medicine, Philadelphia, Pennsylvania, United States of America; Klinikum rechts der Isar der TU München, Germany

## Abstract

Pancreatic ductal adenocarcinoma is believed to arise from precursor lesions termed pancreatic intraepithelial neoplasia (PanIN). Mouse models have demonstrated that targeted expression of activated K-ras to mature acinar cells in the pancreas induces the spontaneous development of PanIN lesions; implying acinar-to-ductal metaplasia (ADM) is a key event in this process. Recent studies suggest Notch signaling is a key regulator of ADM. To assess if Notch1 is required for K-ras driven ADM we employed both an *in vivo* mouse model and *in vitro* explant culture system, in which an oncogenic allele of *K-ras* is activated and *Notch1* is deleted simultaneously in acinar cells. Our results demonstrate that oncogenic *K-ras* is sufficient to drive ADM both *in vitro* and *in vivo* but that loss of Notch1 has a minimal effect on this process. Interestingly, while loss of Notch1 *in vivo* does not affect the severity of PanIN lesions observed, the overall numbers of lesions were greater in mice with deleted Notch1. This suggests Notch1 deletion renders acinar cells more susceptible to formation of K-ras-induced PanINs.

## Introduction

Pancreatic ductal adenocarcinoma (PDAC) is one of the most aggressive forms of human cancer, with a 5-year survival rate of less than 4% [1]. PDAC is believed to arise from precursor lesions termed pancreatic intraepithelial neoplasia (PanIN), which progress through defined stages ultimately leading to the development of adenocarcinoma [2]. The most commonly mutated gene in PDAC is *K-ras*, with greater than 90% of human cases harboring an activating mutation in this oncogene. *K-ras* mutations appear to occur early during the pathogenesis of PDAC, as low-grade PanIN lesions typically contain activating mutations at codon 12 [2]. Further proof that *K-ras* mutations represent an initiating event in PDAC comes from mouse models, in which expression of a mutant activated *K-ras* allele (*K-ras^G12D^*) in pancreatic epithelium is sufficient to induce the formation of both PanIN lesions and invasive pancreatic cancer, pathologically resembling the human disease [3].

The pancreas is composed of an exocrine and endocrine compartment, with the exocrine compartment consisting of acinar, ductal, and centroacinar cells. While the cell of origin for PDAC has remained elusive, recent studies utilizing mouse models have demonstrated that targeting oncogenic *K-ras* to mature acinar cells results in the spontaneous development of PanIN lesions, suggesting acinar cells represent the cell of origin for PDAC [4]. A feature of this model is the appearance of acinar-to-ductal metaplasia (ADM) preceding the development of PanIN lesions. Other studies have highlighted the importance of pancreatic injury in the development of PDAC. Work by Guerra and colleagues revealed that mature acinar cells expressing K-ras^G12V^ are refractory to PanIN development unless mice are subjected to additional stimuli such as chronic chemically-induced pancreatitis [5]. Further, endocrine cells can be made susceptible to oncogenic K-ras induced transformation in the context of pancreatic injury [6]. These findings are especially relevant to human disease, in that chronic pancreatitis is a strong risk factor for the development of PDAC [7].

The Notch signaling family of proteins is composed of 4 transmembrane receptors (Notch1–4), in addition to 2 Jagged ligands, and 3 Delta-like ligands. During pancreatic development, Notch signaling is required for directing cell fate decisions and progenitor cell self renewal [8]. While the role of Notch signaling in development is well characterized, the cell types expressing Notch proteins and their function in the adult pancreas remains unclear. Recent findings indicate Notch1 plays a role in pancreatic homeostasis, since loss of Notch1 in pancreatic epithelium results in impaired acinar regeneration following acute pancreatitis [9]. Moreover, Notch signaling has been implicated in ADM in that ectopic expression of transcriptionally active forms of Notch (Nic) promote transdifferentiation in explant culture models [10]–[11]. Conversely, inhibition of Notch signaling by a γ-secretase inhibitor increases the proliferation of metaplastic exocrine cells and induces p21 expression [12]. Further work demonstrates different Notch receptors have non-overlapping functions and are expressed in unique cellular compartments, with Notch1 observed primarily in acinar cells and Notch2 expressed mainly in ductal cells [13].

Although Notch1 was originally identified as an oncogene, recent evidence indicates the Notch proteins also function as tumor suppressors in a tissue-specific manner. Conclusive evidence demonstrating Notch1 acts as a tumor suppressor came from studies in the skin, where loss of both *Notch1* alleles led to development of basal cell carcinoma [14]. Subsequently, Notch receptors have been identified as tumor suppressors in hepatocellular carcinoma, chronic myelomonocytic leukaemia, and squamous cell carcinomas [15], [16], [17], [18]. Previously unknown loss of function mutations in components of the Notch pathway have been discovered in myeloid leukaemia and squamous cell carcinomas, pointing to a cell autonomous mechanism of tumor suppression for these malignancies. Alternatively, in basal cell carcinoma, Notch1 appears to function in a non-cell autonomous manner by mechanisms impacting the tumor microenvironment [19].

Previous work by our group has identified Notch1 as a tumor suppressor in a mouse model of PDAC [20]. To further investigate the mechanism of Notch1 mediated tumor suppression in pancreatic tumorigenesis we examined the effect of Notch1 deletion on acinar-to-ductal metaplasia both *in vitro* and *in vivo*. These experiments aimed to identify a cell autonomous mechanism of Notch1 mediated tumor suppression. Additionally, we investigated a potential non-cell autonomous function of Notch1 using an orthotopic transplantation tumor model.

## Results

### K-ras Mediated ADM does not Require Notch1 Function

**Figure 1:**
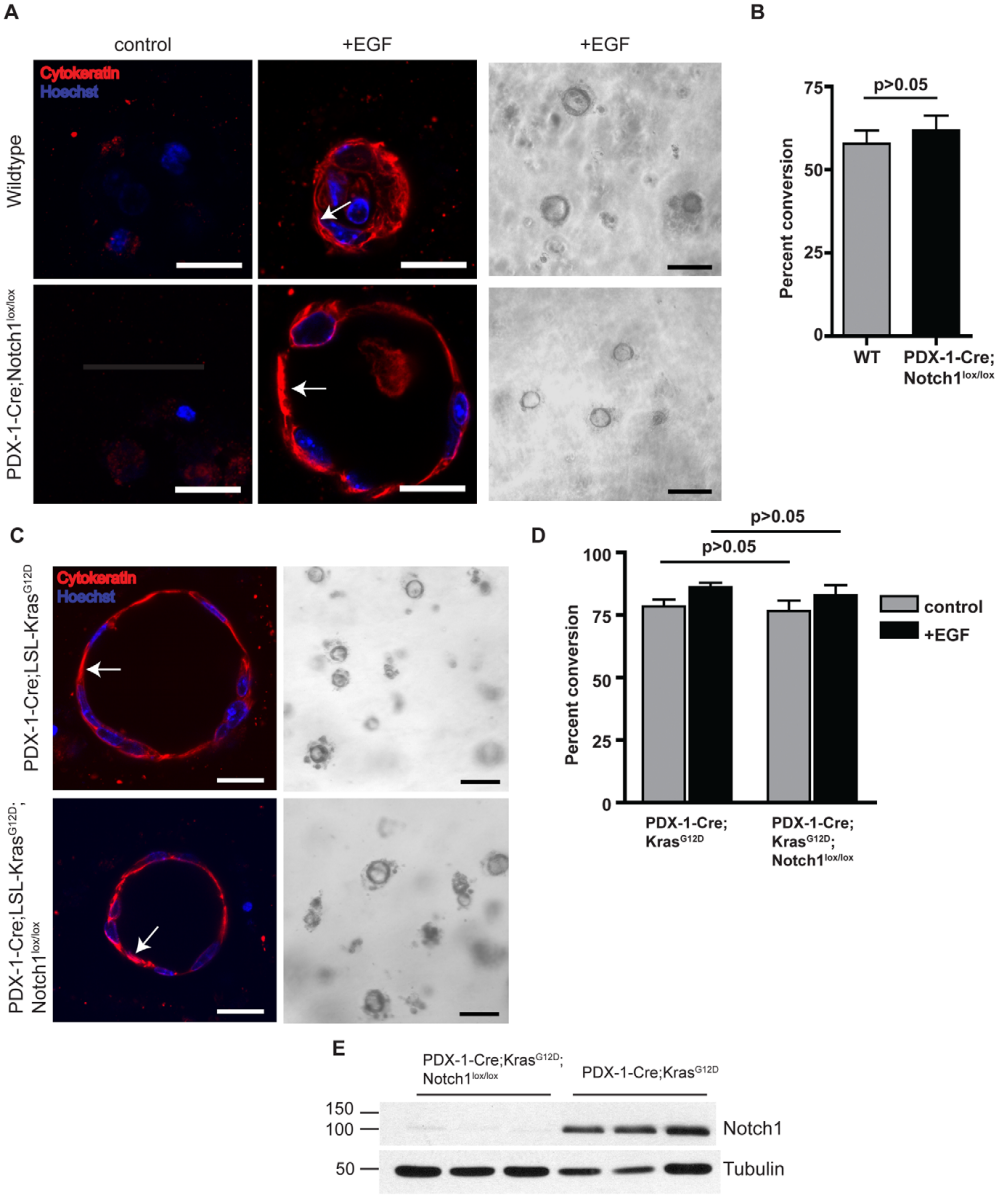
Notch1 is not required for oncogenic K-ras mediated ADM *in vitro*. (**A**) Pancreatic explants from wildtype and *PDX-1-Cre;Notch1^lox/lox^* mice embedded in collagen either untreated (control) or treated with EGF (20 µg/mL). Cells are immunostained for expression of pan-cytokeratin (red) at day 5. Nuclei are stained with Hoechst dye (blue). Scale bar, 20 µm. Arrows indicate cytokeratin-positive ductal cells. Representative brightfield images are shown at day 5 in the presence of EGF. Scale bar, 100 µm. (**B**) Quantitative analysis of percent ductal cyst conversion on day 5 in explants isolated from wildtype and *PDX-1-Cre;Notch1^lox/lox^* mice. n = 3 for each group. (**C**) Pancreatic explants from *PDX-1-Cre;LSL-Kras^G12D^* and *PDX-1-Cre;LSL-Kras^G12D^;Notch1^lox/lox^* mice were isolated at day 2 in the absence of EGF. Cells are immunostained for pan-cytokeratin (red) and Hoechst dye (blue). Scale bar, 20 µm. Arrows indicate cytokeratin-positive ductal cells. Representative brightfield images of cyst formation are shown. Scale bar, 100 µm. (**D**) Quantitative analysis of percent ductal cyst conversion at day 2 in explants isolated from *PDX-1-Cre;LSL-Kras^G12D^* (n = 5) and *PDX-1-Cre;LSL-Kras^G12D^;Notch1^lox/lox^* mice (n = 6). (**E**) Western blot analysis of Notch1 expression in acinar cells isolated from *PDX-1-Cre;LSL-Kras^G12D^* and *PDX-1-Cre;LSL-Kras^G12D^;Notch1^lox/lox^* mice; tubulin as loading control. Three samples are shown for each genotype.

We have recently demonstrated that loss of Notch1 in a mouse model of K-ras-induced PDAC leads to increased PanIN incidence and progression [20]. To further investigate the mechanism of Notch1 mediated PanIN suppression, we examined the role of Notch1 in ADM using an *in vitro* explant culture model. Acinar cell clusters isolated from an adult mouse pancreas transdifferentiate to form cytokeratin-19 positive ductal cysts when embedded in a collagen matrix and treated with growth factors such as EGF or TGF-α [21]. In order to examine the effect of *Notch1* deletion on cyst formation, we utilized *PDX-1-Cre;Notch1^lox/lox^* mice, which allow for conditional deletion of *Notch1^lox/lox^* alleles specifically in pancreatic epithelial cells at day 8.5 of embryonic development [22]. Acinar cells isolated from *PDX-1-Cre;Notch1^lox/lox^* mice formed ductal cysts in the presence of EGF at comparable rates to wildtype acinar cells (Figure 1A and 1B). The acinar origin of the isolated cells was verified by immunostaining for the acinar marker, amylase (Figure S1). These results indicate loss of Notch1 does not accelerate ADM and that Notch1 is not required for EGF-induced ADM *in vitro*.

We next examined whether loss of Notch1 in the context of activated K-ras accelerates acinar-to-ductal conversion *in vitro*. Recently it has been shown that K-ras^G12D^ expression is sufficient to induce pancreatic ADM in explant cultures in the absence of exogenous growth factors [23]. Acinar cells were isolated from *PDX-1-Cre;LSL-Kras^G12D^* and *PDX-1-Cre;LSL-Kras^G12D^;Notch1^lox/lox^* mice. As early as 2 days after isolation, both *PDX-1-Cre;LSL-Kras^G12D^* and *PDX-1-Cre;LSL-Kras^G12D^;Notch1^lox/lox^* acinar explants underwent transdifferentiation to form cytokeratin positive ductal cysts in the absence of external growth factors (Figure 1C), confirming that oncogenic Kras expression is sufficient to drive ADM. Wild type acinar explants failed to undergo transdifferentiation at day 2 even in the presence of EGF (data not shown). Greater than 75% of *PDX-1-Cre;LSL-Kras^G12D^* and *PDX-1-Cre;LSL-Kras^G12D^;Notch1^lox/lox^* acinar explants underwent ADM conversion at day 2. Addition of EGF did not significantly increase rates of conversion in either *PDX-1-Cre;LSL-Kras^G12D^* or *PDX-1-Cre;LSL-Kras^G12D^;Notch1^lox/lox^* explants (Figure 1D).

Previous studies utilizing *PDX-1-Cre;ROSA26R-LacZ* mice have revealed a mosaic recombination pattern in the pancreas [13]. Thus, to confirm that our results do not reflect variations in recombination efficiency of the *Notch1^lox/lox^* allele, we assessed the expression of Notch1 in the explants. Western blot analysis confirmed Notch1 expression in acinar cells isolated from *PDX-1-Cre;LSL-Kras^G12D^* mice and Notch1 deletion in cells isolated from *PDX-1-Cre;LSL-Kras^G12D^;Notch1^lox/lox^* mice (Figure 1E) and reflect our previous findings regarding the recombination of the *Notch1^lox/lox^ in vivo* [20]. Further, rates of ductal cyst formation are very similar between the different samples, indicating similar rates of recombination and activation of the *LSL-Kras^G12D^* allele. Therefore, despite the possibility of mosaic Cre expression, the variability likely does not affect the interpretation of our results.

These results further demonstrate that K-ras^G12D^ expression is sufficient to drive ADM *in vitro* and that Notch1 is not required for ADM in this context.

### The Role of Notch Pathway Signaling in K-ras Induced Acinar-to-ductal Metaplasia

**Figure 2:**
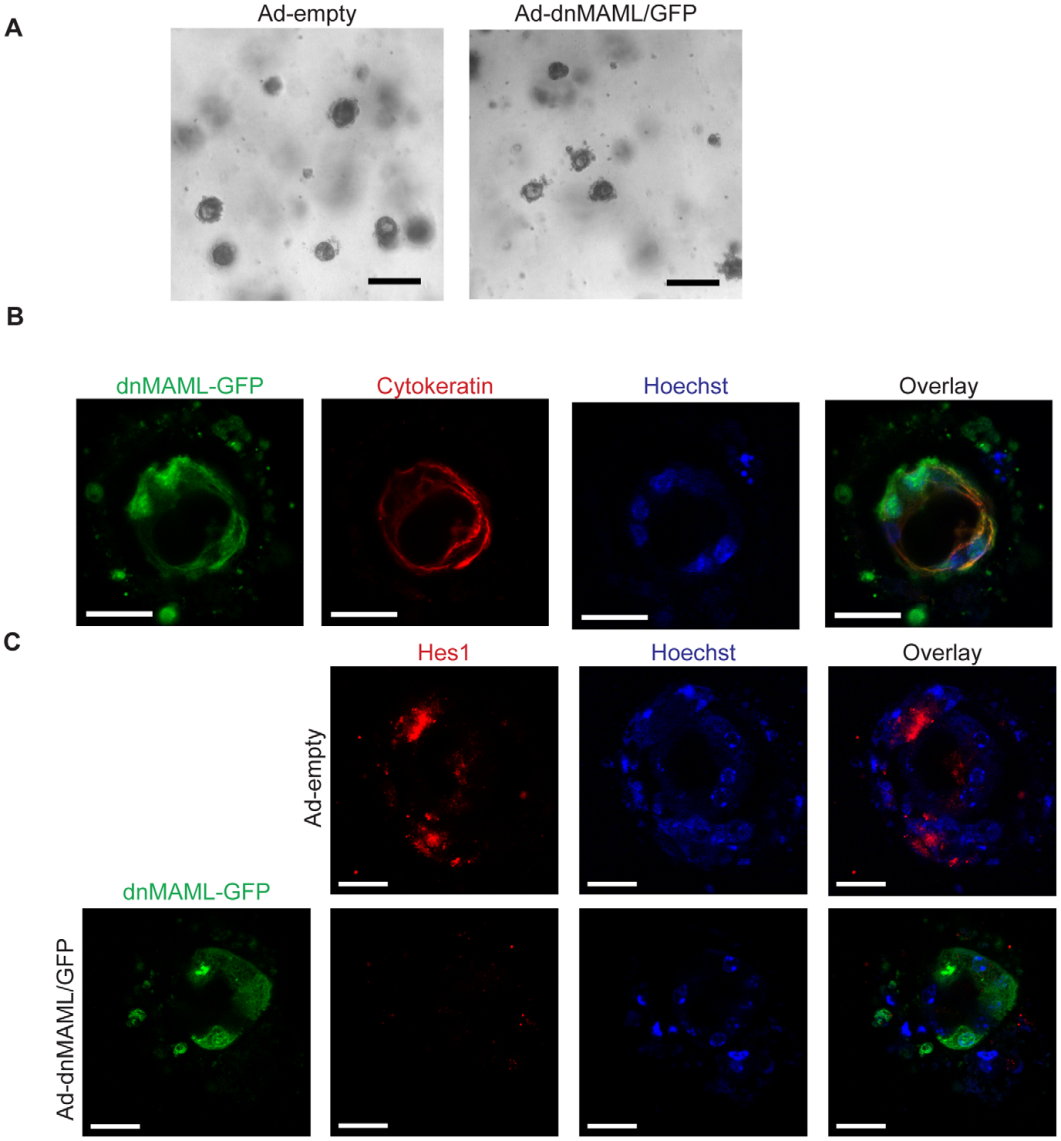
DNMAML expression does not inhibit oncogenic K-ras mediated ADM *in vitro*. (**A**) Pancreatic explants from *Pdx1-Cre;LSL-Kras^G12D^* mice at Day 2, infected with control adenovirus (Ad-empty) or Adenovirus expressing DNMAML (Ad-dnMAML/GFP). Representative brightfield images of ductal cysts shown. Scale bar, 100 µm. (**B**) *Pdx1-Cre;LSL-Kras^G12D^* acinar explants expressing DNMAML-GFP form cytokeratin positive cysts. Explants were stained with antibodies against GFP (green) and pan-cytokeratin (red). Nuclei are counterstained with Hoechst (blue). Images are representative of 3 separate experiments. Scale bar, 20 µm. (**C**) DNMAML expression inhibits Notch signaling in *Pdx1-Cre;LSL-Kras^G12D^* acinar explants. Explants were stained with antibodies against GFP (green) and Hes1 (red). Nuclei are counterstained with Hoechst (blue). Scale bar, 20 µm.

The Notch receptor family contains four members, Notch1–4. Given previous reports suggesting functional overlap between the different receptors, we investigated the effect of inhibiting multiple members of the Notch receptor family simultaneously on K-ras induced ADM *in vitro*. Notch signaling requires the transcriptional co-activator proteins Mastermind-like (MAML1–3) for transcription of downstream target genes. When overexpressed, a truncated form of MAML1 (aa 13–74) acts as a potent dominant-negative mutant, inhibiting Notch-mediated transcriptional activation [24]. We isolated acinar cells from *PDX-1-Cre;LSL-Kras^G12D^* mice and infected them with an adenovirus expressing DNMAML1 fused to GFP. Even in the presence of DNMAML1-GFP, *PDX-1-Cre;LSL-Kras^G12D^* acinar cells maintained the ability to transdifferentiate to cytokeratin positive ductal cysts at day 2, similar to cells expressing a control adenovirus (Figure 2A–B). In order to verify that Notch signaling is inhibited in the presence of DNMAML1, we analyzed expression of Hes1, a downstream effector of Notch signaling. Acinar cells infected with the control adenovirus express Hes1; however, Hes1 expression is reduced when cells are infected with Ad-DNMAML1-GFP (Figure 2C). To further examine the effect of globally inhibiting Notch signaling, we analyzed the effect of a gamma secretase inhibitor, DAPT, on acinar transdifferentiation. *PDX-1-Cre;LSL-Kras^G12D^* acinar cells treated with DAPT maintained the ability to form ctyokeratin positive ductal cysts (Figure S2). Overall, these results indicate that in the presence of oncogenic K-ras, Notch pathway signaling is not required for ADM *in vitro*.

### Deletion of Notch1 does not Accelerate K-ras Induced PanIN Development

**Figure 3:**
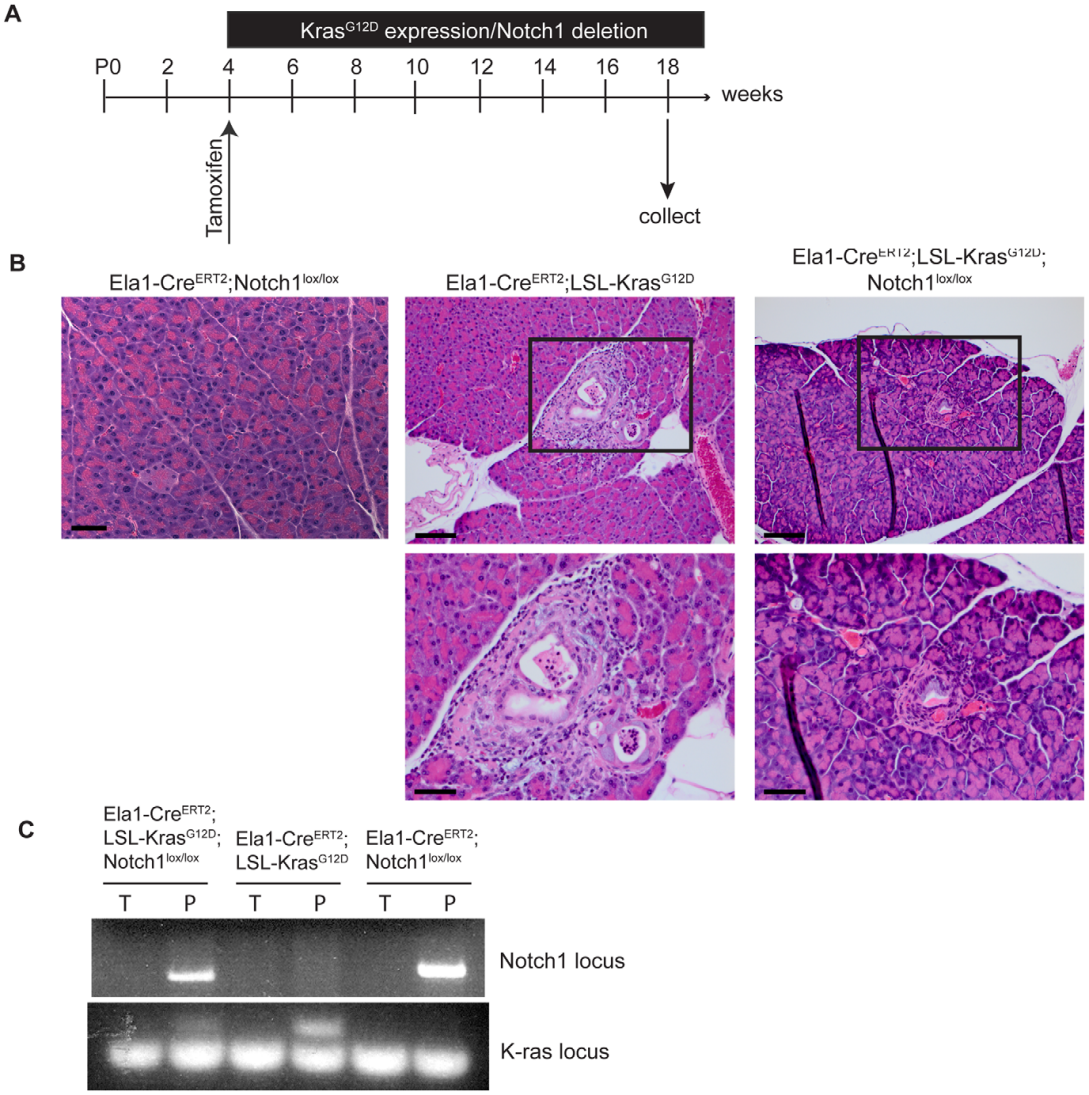
Notch1 deletion in mature acinar cells does not accelerate spontaneous PanIN development. (**A**) Schematic of experimental design. Mice were treated with tamoxifen at 4 weeks of age to activate *K-ras^G12D^* and delete *Notch1* expression. Pancreatic tissue was collected 3 months later. (**B**) Histological analysis of pancreas tissue from *Elastase1-Cre^ERT2^;Notch1^lox/lox^*, *Elastase1-Cre^ERT2^;Kras^G12D^*, and *Elastase1-Cre^ERT2^;Kras^G12D^;Notch1^lox/lox^* mice. Higher magnifications of the boxed areas are seen below the images. Scale bar for top images, 100 µm. Scale bar for higher magnification, 50 µm. (**C**) PCR analysis of Cre-mediated recombination of the *LSL-Kras^G12D^* and *Notch1^lox/lox^* loci. Genomic DNA was isolated from the tail (T) and pancreas (P) of each mouse. For the Notch1 PCR, the presence of a band indicates deletion of the floxed *Notch1* gene. For the *Kras* PCR, the larger band represents deletion of the STOP cassette.

In order to investigate the role of Notch1 in acinar-to-ductal metaplasia *in vivo*, we utilized an *Elastase1-Cre^ERT2^*-driven mouse model. This transgene permits tamoxifen-inducible Cre activation specifically in adult acinar tissue [25]. Activation of oncogenic Kras in the mature acinar compartment results in the spontaneous development of PanIN lesions [4]. Extensive ADM was observed preceding the onset of PanIN lesions in this model, implying acinar cells might represent the cell of origin for PanIN development. *Elastase1-Cre^ERT2^;LSL-Kras^G12D^* and *Elastase1-Cre^ERT2^;LSL-Kras^G12D^;Notch1^lox/lox^* mice were generated, and treated with tamoxifen at 4 weeks of age (Figure 3A). Additionally, *Elastase1-Cre^ERT2^;Notch1^lox/lox^* mice were generated to establish the effect of Notch1 deletion on adult acinar tissue. At 3 months following tamoxifen treatment, pancreas tissue from *Elastase1-Cre^ERT2^;Notch1^lox/lox^* mice appeared grossly normal (Figure 3B). In contrast, pancreas tissue from both *Elastase1-Cre^ERT2^;LSL-Kras^G12D^* and *Elastase1-Cre^ERT2^;LSL-Kras^G12D^;Notch1^lox/lox^* demonstrated mild to moderate fibroplasia and fibrosis. Additionally, low-grade PanIN1A lesions were observed in 1 of 6 *Elastase1-Cre^ERT2^;LSL-Kras^G12D^* mice and 1 of 7 *Elastase1-Cre^ERT2^;LSL-Kras^G12D^;Notch1^lox/lox^* mice (Figure 3B, Figure S3A). Analysis by PCR demonstrated recombination at both the *Kras* and *Notch1* gene loci in the pancerata of these mice (Figure 3C). These results demonstrate that in the context of activated Kras, deletion of *Notch1* in adult acinar tissue does not accelerate spontaneous PanIN development. Additionally, the results suggest that Notch1 is not required for K-ras induced PanIN development, as an *Elastase1-Cre^ERT2^;LSL-Kras^G12D^;Notch1^lox/lox^* mouse developed a PanIN1A lesion. However further studies, with larger animal cohorts, are required to conclusively establish this point and to determine whether Notch1 deletion renders acinar cells more susceptible to K-ras induced transformation, in line with previous studies [20].

### Notch1 Deletion does not Accelerate PanIN Development Following Acute Pancreatitis but Renders Cells More Susceptible to Formation of K-ras-induced PanINs

**Figure 4:**
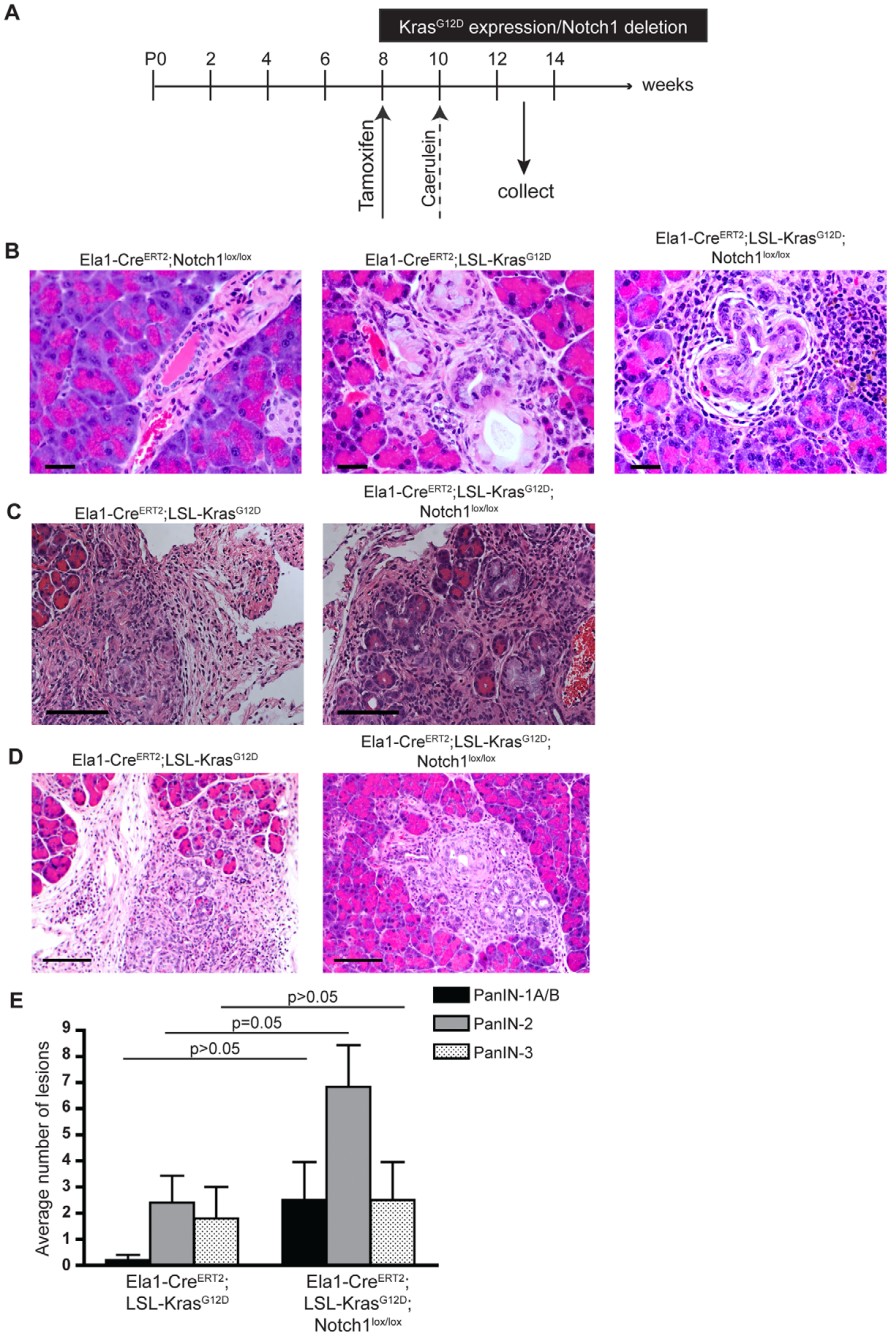
Notch1 deletion does not alter PanIN development following acute pancreatitis. (**A**) Schematic of experimental design. Mice were treated with tamoxifen at 8 weeks of age to activate *K-ras^G12D^* and delete *Notch1* expression. Two weeks later, mice were treated with caerulein for 2 consecutive days to induce acute pancreatitis. Pancreatic tissue was collected 3 weeks following the final caerulein treatment. (**B**) Histological analysis of pancreas tissue from *Elastase1-Cre^ERT2^;Notch1^lox/lox^*, *Elastase1-Cre^ERT2^;Kras^G12D^*, and *Elastase1-Cre^ERT2^;Kras^G12D^;Notch1^lox/lox^* mice following acute pancreatitis. PanIN-3 lesions are shown in *Elastase1-Cre^ERT2^;Kras^G12D^* and *Elastase1-Cre^ERT2^;Kras^G12D^;Notch1^lox/lox^* pancreas tissue. Scale bar, 30 µm. (**C**) Fibroplasia and inflammation observed in *Elastase1-Cre^ERT2^;Kras^G12D^* and *Elastase1-Cre^ERT2^;Kras^G12D^;Notch1^lox/lox^* pancreas tissue. Scale bar, 100 µm. (**D**) Atypical flat lesions (AFLs) were observed in *Elastase1-Cre^ERT2^;Kras^G12D^* and *Elastase1-Cre^ERT2^;Kras^G12D^;Notch1^lox/lox^* pancreas tissue. Scale bar, 100 µm. (**E**) Analysis of the number of PanIN lesions found per pancreas in *Elastase1-Cre^ERT2^;Kras^G12D^* (n = 5) and *Elastase1-Cre^ERT2^;Kras^G12D^;Notch1^lox/lox^* (n = 6) following acute pancreatitis.

As our results demonstrated a comparatively mild phenotype in *Elastase1-Cre^ERT2^;LSL-Kras^G12D^* and *Elastase1-Cre^ERT2^;LSL-Kras^G12D^;Notch1^lox/lox^* mice, we proceeded to investigate the effect of caerulein-induced pancreatitis. Previous studies have demonstrated that oncogenic K-ras expression in adult acinar cells is sufficient to drive PanIN formation [4], [26], yet the process is highly inefficient. Moreover, similar mouse models have revealed that adult acinar cells are refractory to PanIN development unless mice are subjected to caerulein-induced pancreatitis [5]. Therefore, we compared pathology between *Elastase1-Cre^ERT2^;Notch1^lox/lox^*, *Elastase1-Cre^ERT2^;LSL-K-ras^G12D^* and *Elastase1-Cre^ERT2^;LSL-K-ras^G12D^;Notch1^lox/lox^* mice following tamoxifen induction and acute pancreatitis (Figure 4A). Three weeks following caerulein treatment, the pancreas from *Elastase1-Cre^ERT2^;Notch1^lox/lox^* mice appeared grossly normal (Figure 4B). Similar to previous models, pancreas tissue from *Elastase1-Cre^ERT2^;LSL-K-ras^G12D^* (n = 5) mice displayed a range of PanIN lesions, with PanIN-3 being the most severe (Figure 4B, Figure S3B). Additionally, moderate fibroplasia and inflammation were noted in all samples (Figure 4C), as well as atypical flat lesions (AFLs), which arise in areas of acinar-to-ductal metaplasia (Figure 4D) [27]. Similar grades of pancreatic pathology were noted in *Elastase1-Cre^ERT2^;LSL-K-ras^G12D^;Notch1^lox/lox^* (n = 6) mice, indicating Notch1 deletion does not accelerate PanIN formation following acute pancreatitis (Figure 4B and C). However, further analysis to determine the prevalence of the different lesions revealed that there was a trend for the *Elastase1-Cre^ERT2^;LSL-K-ras^G12D^;Notch1^lox/lox^* mice to develop a greater number of AFLs and PanIN lesions (Figure 4D and 4E). The average number of PanIN1-A/B, PanIN-2, and PanIN-3 lesions was greater in *Elastase1-Cre^ERT2^;LSL-K-ras^G12D^;Notch1^lox/lox^* mice; however, only the difference between PanIN-2 lesions was statistically significant. Additionally, only 1 of 5 *Elastase1-Cre^ERT2^;LSL-K-ras^G12D^* mice developed AFLs, while 4 of 6 *Elastase1-Cre^ERT2^;LSL-K-ras^G12D^;Notch1^lox/lox^* mice displayed the lesions. These results suggest Notch1 deletion renders acinar cells more susceptible to formation of K-ras-induced PanIN lesions and AFLs.

### Notch1 Functions in a Cell Autonomous Manner to Inhibit Tumorigenesis

**Figure 5:**
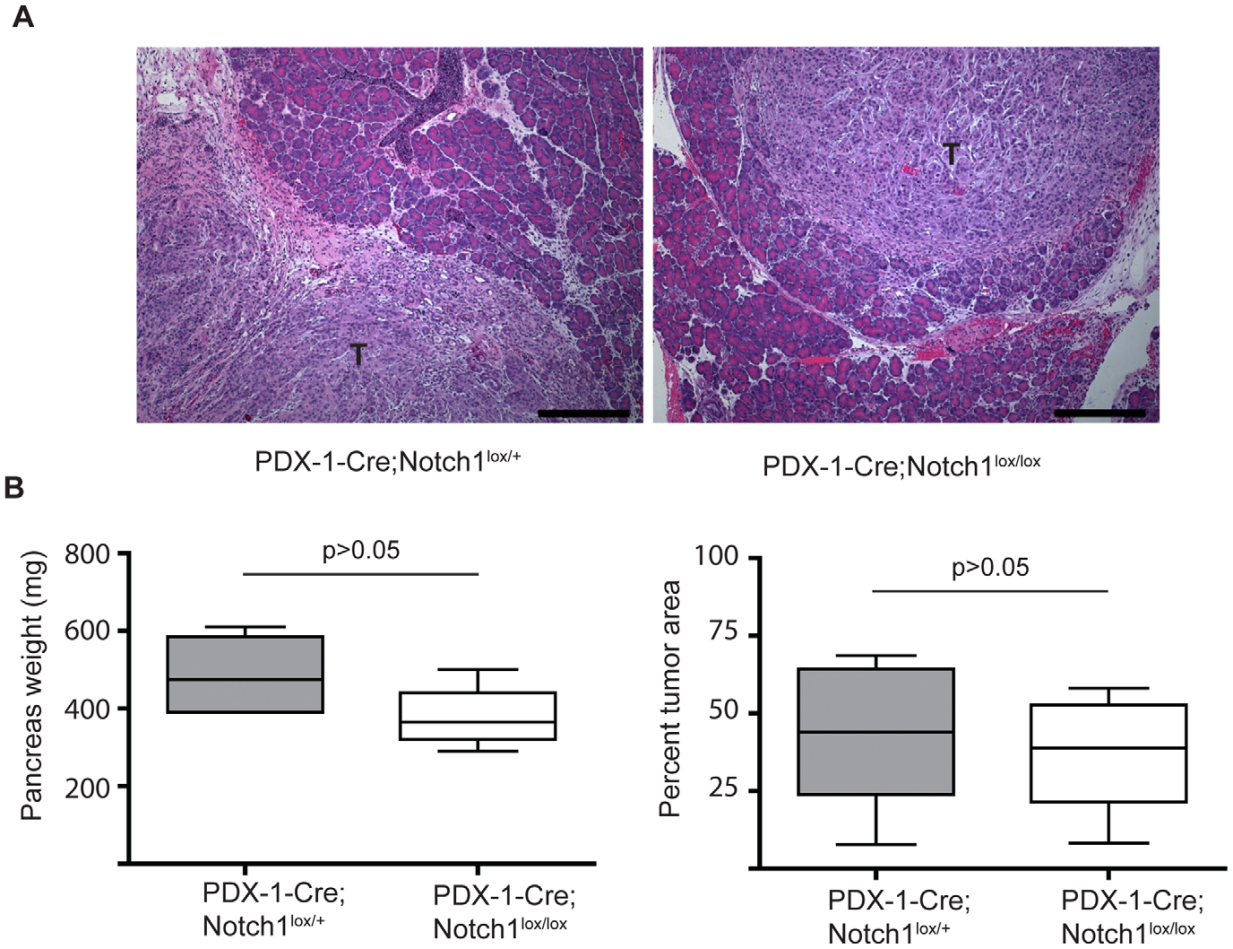
Notch1 functions in a cell autonomous manner. (**A**) Representative images of orthotopic tumors in *Pdx1-cre;Notch^lox/+^* and *Pdx1-Cre;Notch1^lox/lox^* mice. Tumor cells (T) are seen adjacent to normal pancreatic tissue. Scale bar, 300 µm (**B**) Quantitative analysis of pancreas weight and percentage of tumor volume in *Pdx1-Cre;Notch^lox/+^*(n = 5) and *Pdx1-Cre;Notch1^lox/lox^* (n = 6) mice 2 weeks after orthotopic injections. Box plots depict median values, lower and upper quartiles, and maximum and minimum observed values.

Previous studies have demonstrated that Notch1 functions as a tumor suppressor gene in the skin by mechanisms impacting the tumor microenvironment [19]. In addition, it has recently been shown that treatment with a γ-secretase inhibitor has a greater impact on the destabilization of endothelial cells rather than primary tumors in a mouse model of pancreatic cancer [28]. These studies indicate Notch proteins may function primarily in a non-cell autonomous manner in the tumor environment. To determine whether Notch1 functions in a non-cell autonomous mechanism to inhibit pancreatic tumorigenesis, we employed an orthotopic transplantation model of tumorigenesis. *PDX-1-Cre;Notch1^lox/lox^* and *PDX-1-Cre;Notch1^lox/+^* mice were crossed to NOD scid gamma mice to generate immunodeficient hosts for transplantation. Pancreatic epithelial cell lines isolated from primary tumors of *PDX-1-Cre;LSL-Kras^G12D^;LSL-Trp53^R172H^* (KPC) mice [29] were injected into the pancreas of either *PDX-1-Cre;Notch1^lox/lox^* (Notch absent) or *PDX-1-Cre;Notch1^lox/+^* (Notch present) mice. It should be noted that the *PDX-1-Cre;Notch1^lox/lox^* mice have Notch1 deleted from all pancreatic epithelial cells; however, other cells located in the tumor microenvironment, such as endothelial cells and immune cells, retain Notch1 expression. After 2 weeks, KPC cells implanted into both *PDX-1-Cre;Notch1^lox/lox^* and *PDX-1-Cre;Notch1^lox/+^* mice formed palpable tumors (Figure 5A). Significant differences were not found in either pancreas weight or percent tumor area between the *PDX-1-Cre;Notch1^lox/lox^* and *PDX-1-Cre;Notch1^lox/+^* hosts (Figure 5B). These results indicate Notch1 does not function in a non-cell autonomous manner to inhibit PDAC tumor maintenance.

## Discussion

Evidence now suggests PanIN lesions, and ultimately PDAC, can arise from acinar to ductal metaplasia [30]. The exact molecular mechanisms controlling the conversion of normal acinar tissue to metaplastic lesions remains unclear, however activation of oncogenic K-ras appears to be a key event in the transdifferentiation process. Similar to a previous report [23], we find that expression of *K-ras^G12D^* is sufficient to induce acinar to ductal metaplasia in an *in vitro* model. This finding supports animal models in which activation of oncogenic K-ras in pancreatic acinar cells induces PanIN development, and provides further evidence that *K-ras* mutations are an initiating event in the development of PDAC.

Previous reports have suggested Notch signaling is activated in PanIN lesions and PDAC in humans [10] and in mouse models [4]. Further, inhibition of Notch signaling by treatment with a γ-secretase inhibitor (GSI) prevents tumor progression in a mouse model of PDAC [31]. Given that Notch receptors regulate cell fate decisions during pancreatic development, it is reasonable to assume Notch signaling is activated during ADM. Accordingly, ectopic expression of the activated forms of Notch1 or Notch2 (Nic) drives ADM in *in vitro* explant culture models [10], [11].

In contrast to these reports, previous work by our lab has demonstrated Notch1 suppresses PanIN formation in a mouse model of PDAC [20]. In the current study, we investigated the requirement for Notch1 in ADM using an explant culture model. Surprisingly, we find that Notch1 is not required for either EGF- or K-ras^G12D^-mediated transdifferentiation. In addition, using a dominant-negative form of MAML1 as a pan-Notch inhibitor, we show that Notch signaling is not required for ADM *in vitro*. However, it should be noted that MAML1 also functions as a transcriptional activator independent of the Notch signaling pathway [32]. The possibility therefore remains that Notch signaling may be functioning independent of MAML in order to regulate ADM *in vitro*.

One potential explanation for the differences between our results and previous reports indicating Notch can promote ADM is the fact that previous studies employed ectopic expression of activated forms of Notch receptors, leading to expression of Nic above physiological levels. Thus, the contrasting outcomes observed when Notch1 is genetically deleted versus overexpressed can be attributed to different phenotypic responses to levels of pathway activation. Indeed this has been demonstrated in studies showing that Nic expression levels affect the balance between growth-stimulating and growth-suppressive effects in mammary epithelial cultures [33]. An additional factor to consider is that many earlier studies rely on Hes1 expression as a surrogate for Notch activation, while we and others have found that inhibition of Notch signaling in the pancreas does not reduce Hes1 expression [12], [13]. Further, ectopic expression of Hes1 in an *in vitro* ADM assay fails to recapitulate the effects of Nic expression, implying additional downstream effectors are responsible for Notch mediated events [10]. Hence, it will be beneficial to identify all downstream effectors of Notch signaling specific to the different cellular compartments of the pancreas.

To further assess the role of Notch1 in ADM *in vivo*, we employed the *Elastase1-Cre^ERT2^* model to drive *K-ras^G12D^* expression and *Notch1* deletion. Unlike the *PDX-1-Cre* transgene, which induces Cre-mediated recombination during embryonic development, the *Elastase1-Cre^ERT2^* model allows for the activation of Cre and recombination in mature acinar cells. Using this model we found that deletion of *Notch1* in the context of activated *K-ras* does not accelerate the development of PanIN lesions. These results differ from our previous work using *PDX-1-Cre;LSL-Kras^G12D^* mice, where we found deletion of *Notch1* accelerated PanIN formation. One reason for the differences in the two models is the timing of *Notch1* deletion. In the *PDX-1-Cre* model, *Notch1* is deleted at embryonic day 8.5 (e8.5) during a crucial stage of pancreatic development. Though Notch1 deletion at this stage does not cause defects in pancreatogenesis [34], progenitor cells may become more susceptible to oncogene induced transformation upon *Notch1* deletion. Additionally, the timing of genetic events during tumor progression may be crucial. In human pancreatic tumors, *K-ras* mutations occur at the earliest stages of PanIN development, while loss of commonly mutated tumor suppressor genes, such as p53 and INK4a occurs later in the PanIN-PDAC lineage [7]. The chronological order in which these events occur in mouse models may be critical to tumor development and progression. Therefore it is possible that *Notch1* deletion during development, concurrently with *K-ras^G12D^* activation, leads to a different phenotype than deletion of *Notch1* in the mature pancreas.

Alternatively, the seeming discrepancy in these results may stem from Notch receptors being expressed in specific cellular compartments. Whereas the *PDX-1-Cre* allele deletes *Notch1* in all pancreatic epithelial cells, the *Elastase1-Cre^ERT2^* model deletes *Notch1* exclusively in acinar cells. The results of both our *in vitro* and *in vivo* experiments demonstrate loss of Notch1 does not accelerate spontaneous ADM, indicating Notch1 may function in a different cellular compartment. One possible cell type to consider is centroacinar cells (CACs) which lie at the junction of the duct network and acini and which express Hes1 [35]. Recent work using a *Hes1^CreERT2^* model identified CACs, and also a smaller fraction of ductal cells, as Notch responsive cells in the pancreas [36]. Future studies are needed to elucidate how genetic deletion of *Notch1* specifically in these populations affects PanIN development and if these results support our findings in the *PDX-1-Cre* model.

We further investigated the effect of Notch1 deletion in the *Elastase1-Cre^ERT2^;LSL-K-ras^G12D^* model following acute pancreatitis. Recent studies indicate that acinar cell regeneration is inhibited in response to both oncogenic K-ras activation and Notch1 deletion [7], [9]. Our results suggest that Notch1 deletion in the *Elastase1-Cre^ERT2^* model does not affect acinar regeneration, as pancreas tissue from *Elastase1-Cre^ERT2^;Notch1^lox/lox^* mice appeared grossly normal 3 months following caerulein treatment. However, we did not examine earlier time points after injury, when acinar regeneration is most pronounced. These opposing results may also be explained by the use of different Cre-drivers, as previous studies utilized *Ptf1a^+/Cre(ex1)^* rather than an acinar-specific promoter. As the *Ptf1a^+/Cre(ex1)^* line targets a greater number of acinar cells compared to *Elastase1-Cre^ERT2^* mice, this may help explain the lack of phenotype in our model.

Following acute pancreatitis, both *Elastase1-Cre^ERT2^;LSL-K-ras^G12D^* and *Elastase1-Cre^ERT2^;LSL-K-ras^G12D^;Notch1^lox/lox^* mice develop high grade PanIN lesions. While the grade of PanIN lesions is similar in both cohorts, the *Elastase1-Cre^ERT2^;LSL-K-ras^G12D^;Notch1^lox/lox^* mice appear to have a greater abundance of PanIN lesions, as well as atypical flat lesions (AFLs), which arise in regions of ADM [27]. These results imply Notch1 deletion renders acinar cells more susceptible to K-ras induced PanIn formation, as well as AFL formation, which may represent an alternative precursor lesion to PanINs. Additional work is needed to determine the relationship between AFLs and the PanIN-PDAC progression model. Further, it remains to be determined how pancreatitis and Notch1 loss potentially synergize to promote oncogenic K-ras induced PanIN formation. However, recent studies examining the effects of oncogenic K-ras activation and Notch1 deletion individually in response to caerulein-induced pancreatitis have provided mechanistic clues. Following acute pancreatitis, acinar cells expressing oncogenic K-ras undergo a state of persistant dedifferentiation, ultimately leading to ADM and PanIN lesion formation [7]. Similarly, mice deficient for Notch1 in pancreatic epithelium also display impaired acinar regeneration following acute pancreatitis [9]. Therefore, activation of K-ras and deletion of Notch1 may cooperate to inhibit the regeneration process, resulting in a population of cells more susceptible to PanIN formation. Interestingly, both of the previous studies identified alterations in β-catenin signaling as a downstream effect of either K-ras^G12D^ activation or Notch1 deletion.

Finally, we investigated whether Notch1 functions through a non-cell autonomous mechanism to suppress tumor maintenance using an orthotopic transplantation model. We investigated a role for Notch1 in the tumor environment by orthotopically injecting KPC tumor cells directly into the pancreas of a mouse in which Notch1 had been deleted in all epithelial cells. Our results suggest Notch1 functions in a cell autonomous manner to inhibit PanIN formation. Further work is needed determine if Notch1 can function to suppress PanIN progression in a non-cell-autonomous manner, as it has been previously shown to function in the skin [19].

In conclusion, our data reveal Notch1 is not required for pancreatic ADM. Additionally, loss of Notch1 in adult acinar cells does not accelerate ADM either *in vitro* or in an *in vivo* mouse model of PDAC, suggesting Notch1 regulates alternative molecular events in PanIN development.

## Materials and Methods

### Mouse Strains

The LSL-Kras^G12D^ [37], Notch1^lox/lox^ [38], Pdx1-Cre [3], and Elastase1-Cre^ERT2^ [25] mouse strains have been previously described. To initiate Cre-mediated recombination in Elastase1-Cre^ERT2^;LSL-Kras^G12D^, Elastase1-Cre^ERT2^;LSL-Kras^G12D^;Notch1^lox/lox^, and Elastase1-Cre^ERT2^;Notch1^lox/lox^ strains, mice were treated with tamoxifen (Sigma-Aldrich) at 4 weeks of age. Mice received 5 consecutive daily intraperitoneal injections of 2 mg of tamoxifen dissolved in sunflower seed oil (Sigma-Aldrich). For pancreatitis experiments, mice were treated with tamoxifen at 8 weeks of age by oral gavage. Three doses were administered (20 mg, 20 mg, 10 mg) over the course of 1 week. Two weeks later, pancreatitis was induced by 6 hourly intraperitoneal injections of caerulein (Sigma-Aldrich), 2 µg dissolved in 0.9% NaCl, over 2 consecutive days. NOD scid gamma (NSG) mice were purchased from Jackson labs, stock number 005557. All mice used were on a mixed background. All studies were approved and conducted in compliance with Wistar Institute Institutional Animal Care and Use Committee guidelines.

### Primary Acinar Cultures

Primary acinar cells were prepared as previously described [21]. Cells were maintained in RPMI1640 media (Cellgro) supplemented with Soybean Trypsin Inhibitor (Sigma-Aldrich) at 0.1 mg/mL, Dexamethazone (1 µg/mL) (Sigma-Aldrich) and Penicillin/Streptomycin (Gibco Life Technologies). When necessary, cells were treated with EGF, 20 ng/mL (BD Biosciences), DAPT, 10 µM (Sigma), or 0.1% DMSO (Sigma). Cultures were maintained at 37°C and 5% CO_2_ for up to 5 days. Media was changed daily. Brightfield images were captured on a Nikon TE2000 inverted microscope. For quantitative analysis, ductal cysts were counted from 20 different focal planes for each sample and divided by the total number of cell aggregates.

### Immunofluorescence

Collagen disks were fixed and rehydrated as previously described [21]. Disks were blocked in PBSBT (PBS, 0.1% tritonX-100, 2%BSA) and subsequently incubated overnight in primary antibodies diluted in PBSBT. Primary antibodies used were anti-cytokeratin (Dako, 1∶500), anti-GFP-Alexa488 conjugate (Invitrogen, 1∶400), and anti-Hes1 (gift from Dr. Ben Stanger, University of Pennsylvania, 1∶100). For cytokeratin and Hes1 staining, disks were washed in PBSBT and incubated overnight in anti-rabbit Alexa568 secondary antibody (Invitrogen, 1∶500). Hoechst 33342 (Sigma-Aldrich) was used to counterstain nuclei. Images were captured on a Zeiss LSM710 Confocal microscope.

### Adenoviral Infection

A cDNA coding for DNMAML-GFP fusion protein (gift from Dr. Warren Pear, University of Pennsylvania) was cloned into an Adenoviral vector. Adenoviral empty vector was used as a control. Prior to plating in collagen, acinar cell suspensions were incubated with adenovirus (MOI 20∶1) at 37°C for 1 hr with occasional rocking. Suspensions were then plated in collagen.

### Western Blot Analysis

Acinar cells were homogenized in RIPA buffer, separated by SDS-PAGE and transferred to PVDF membranes (Immobilon-P, Millipore). Primary antibodies employed were: Notch1 (Epitomics, 1∶500) and Tubulin (Sigma-Aldrich, 1∶10,000). Blots were developed using horseradish peroxidase (HRP)-conjugated secondary antibodies and the ECL detection system (Amersham).

### Histological Analysis and Immunohistochemistry

Formalin-fixed paraffin-embedded pancreatic tissue was processed by standard methods and stained with hematoxylin and eosin (H&E) or immunostained with an antibody for Muc-5AC Glycoprotein (Novocastra, 1∶50 dilution). Antigen retrieval was performed in 10 mM sodium citrate buffer pH 6.0. Labeling was detected using the VECTOR M.O.M. Immunodetection Kit (Vector Laboratories) following the manufacturers protocol. Slides were counterstained with hematoxylin, dehydrated, and mounted. Pancreatic pathology and scoring of lesions were performed by a board-certified veterinary pathologist. Tissue was fixed and embedded in paraffin. For each case, an H&E stained longitudinal section spanning the entire pancreas was analyzed at 20X and 60X magnification.

### *Kras^G12D^* and *Notch1* Allele Recombination PCR

Genomic DNA was isolated from tails and pancreas using a Genelute Mammalian Genomic DNA kit (Sigma-Aldrich). The *LSL-Kras^G12D^* allele [37] and the *Notch1* allele [39] were analyzed by PCR as previously described. The primers for the *LSL-Kras^G12D^* allele were: 5′ GGG TAG GTG TTG GGA TAG CTG and 3′ TCC GAA TTC AGT GAC TAC AGA TGT ACA GAG. The primers used for the *Notch1* allele were: 5′ of loxP1 CTG ACT TAG TAG GGG GAA AAC; 3′ of loxP1 AGT GGT CCA GGG TGT GAG TGT; and 3′ of loxP2 TAA AAA GCG ACA GCT GCG GAG.

### Orthotopic Transplantation Experiments

KPC cells lines were derived from primary tumors of *Pdx1-Cre;LSL-Kras^G12D^;LSL-Trp53^R172H^* mice (gift from Dr. Robert Vonderheide, University of Pennsylvania). Mice were anesthetized in isoflurane and the pancreas was exposed by a lateral incision. 1×10^5^ cells were suspended in 50 µL DMEM (Gibco Life Technologies) and injected into the head of the pancreas using an insulin syringe. The pancreas was returned to the peritoneal cavity, and the peritoneum was closed with 4–0 chromic sutures and the skin was closed with 6–0 silk sutures (Roboz). After 2 weeks the mice were sacrificed and the pancreas was removed and weighed. Tumor to pancreas volume was assessed using ImagePro Plus (Media Cybernetics).

### Statistical Analysis

Data presented is mean±standard error of mean (SEM). Statistical analysis was performed using two-tailed unpaired Student's t test. A p value of equal or less than 0.05 was considered significant.

## Supporting Information

Figure S1 **Immunofluorescence confirms acinar origin of cells. (A)** Pancreatic explants from wildtype mice embedded in collagen either untreated (control) or treated with EGF (20 µg/mL). Cells are immunostained for expression of the acinar marker, amylase (green) and the ductal marker, pan-cytokeratin (red) at day 5. Scale bar, 75 µm.
(TIF)

Figure S2 **DAPT treatment does not inhibit oncogenic K-ras mediated ADM** ***in vitro*** **. (A)** Pancreatic explants from *PDX-1-Cre;LSL-Kras^G12D^* mice embedded in collagen either treated with 0.1% DMSO (control) or DAPT (10 µM). Cells are immunostained at Day 2 for expression of the ductal marker, pan-cytokeratin (red), and counterstained with Hoechst dye. Scale bar, 20 µm.
(TIF)

Figure S3 **Muc-5AC staining identifies PanIN lesions. (A)** Expression of Muc-5AC by immunohistochemical staining in PanIN lesions from *Elastase1-Cre^ERT2^;Kras^G12D^* and *Elastase1-Cre^ERT2^;Kras^G12D^;Notch1^lox/lox^* mice, 3 months following tamoxifen treatment. Scale bar, 50 µm. **(B)** Expression of Muc-5AC by immunohistochemical staining in PanIN lesions from *Elastase1-Cre^ERT2^;Kras^G12D^* and *Elastase1-Cre^ERT2^;Kras^G12D^;Notch1^lox/lox^* mice following tamoxifen treatment and caerulin-induced pancreatitis. Scale bar, 50 µm.
(TIF)
