# Supplementary figures and images for: Notch1 Is Not Required for Acinar-to-Ductal Metaplasia in a Model of Kras-Induced Pancreatic Ductal Adenocarcinoma
Source: PLoS One. 2012 Dec 19;7(12):e52133. doi: 10.1371/journal.pone.0052133 (PMC3526595; doi:10.1371/journal.pone.0052133)

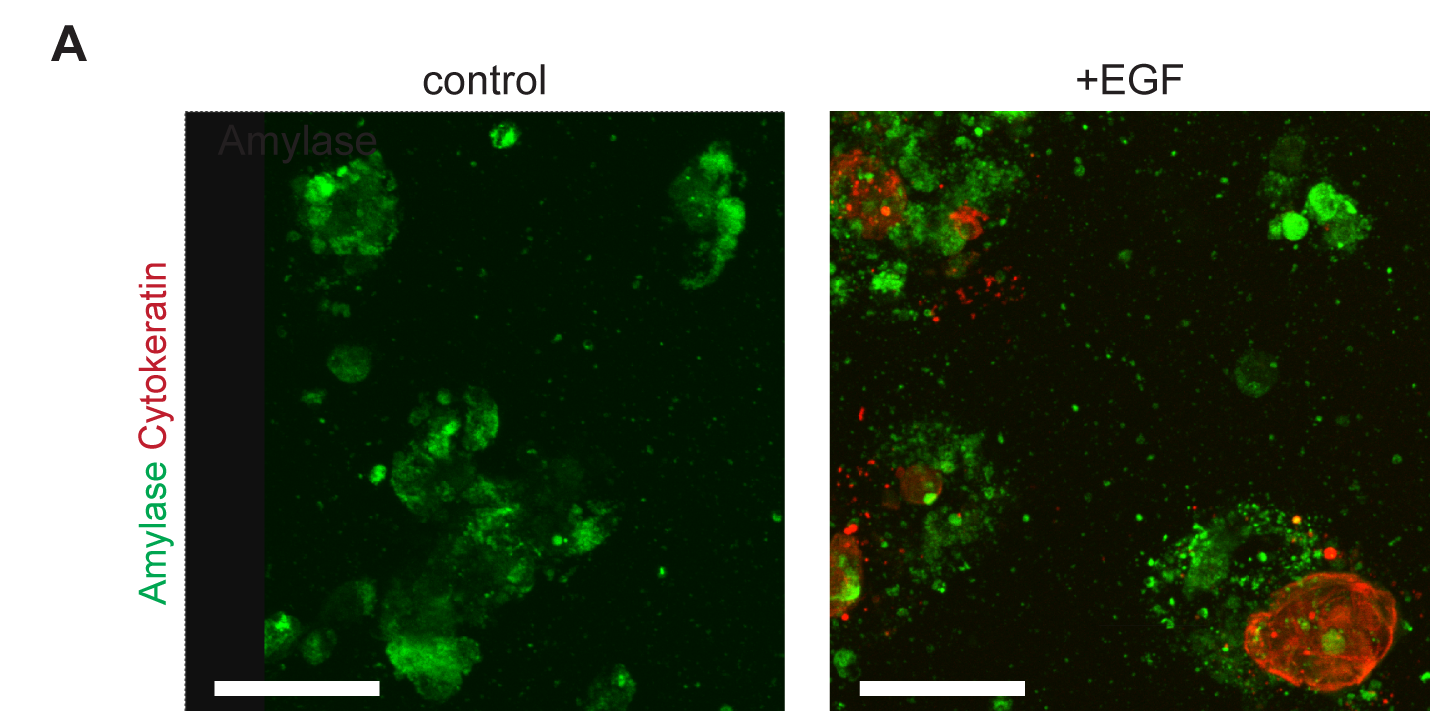

Supplement: Figure S1 — Immunofluorescence confirms acinar origin of cells. (A) Pancreatic explants from wildtype mice embedded in collagen either untreated (control) or treated with EGF (20 µg/mL). Cells are immunostained for expression of the acinar marker, amylase (green) and the ductal marker, pan-cytokeratin (red) at day 5. Scale bar, 75 µm. (TIF) [file pone.0052133.s001.tif]

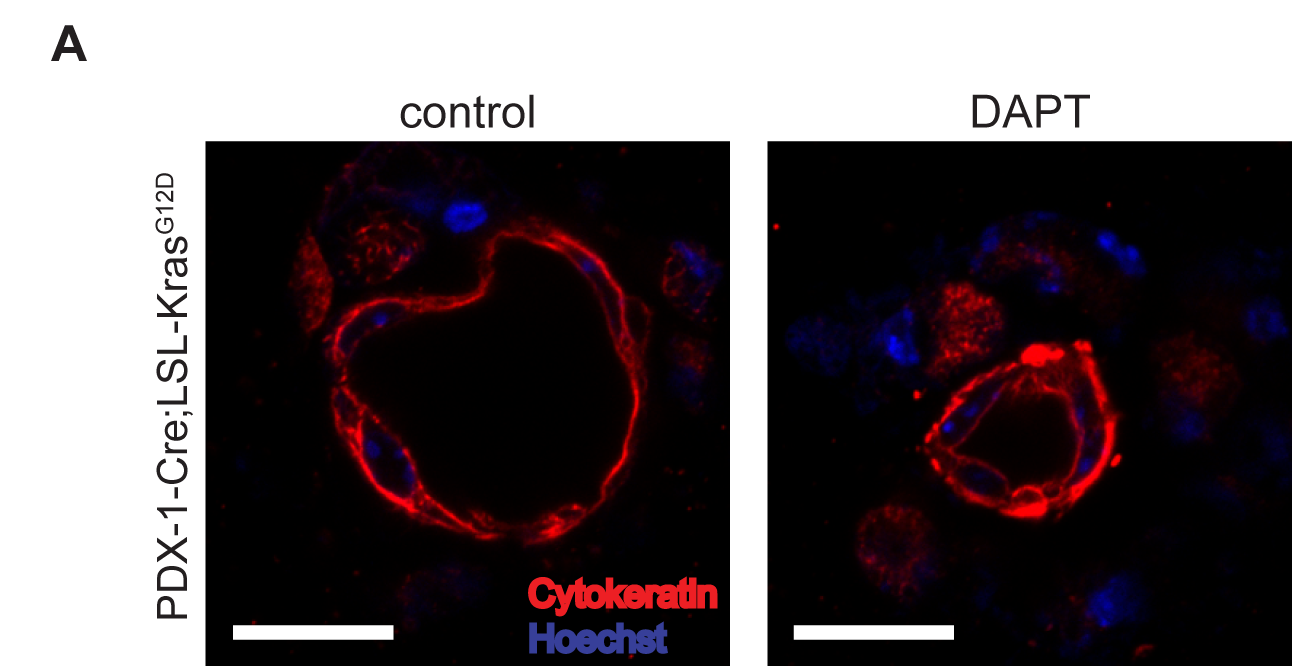

Supplement: Figure S2 — DAPT treatment does not inhibit oncogenic K-ras mediated ADM in vitro . (A) Pancreatic explants from PDX-1-Cre;LSL-KrasG12D mice embedded in collagen either treated with 0.1% DMSO (control) or DAPT (10 µM). Cells are immunostained at Day 2 for expression of the ductal marker, pan-cytokeratin (red), and counterstained with Hoechst dye. Scale bar, 20 µm. (TIF) [file pone.0052133.s002.tif]

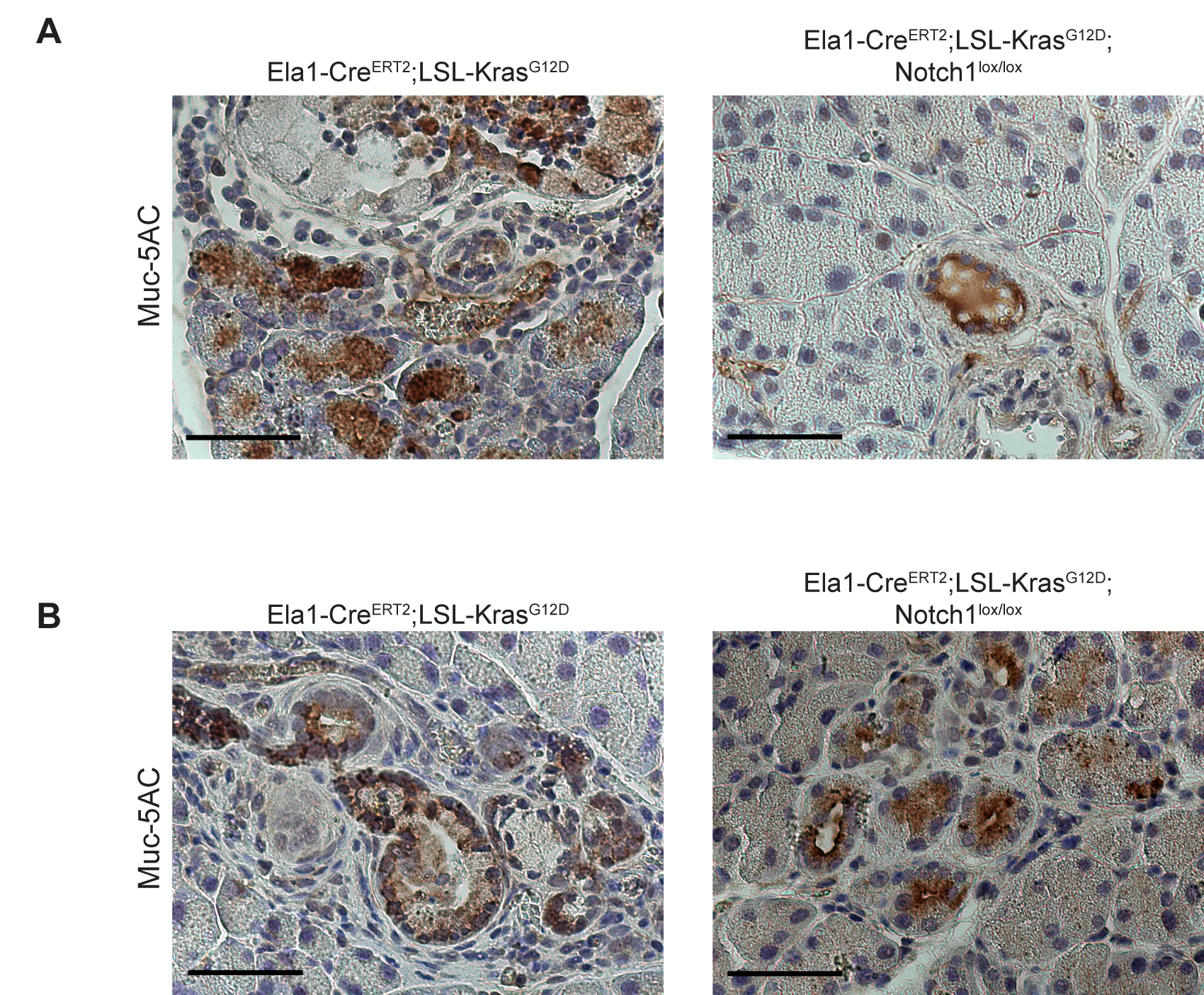

Supplement: Figure S3 — Muc-5AC staining identifies PanIN lesions. (A) Expression of Muc-5AC by immunohistochemical staining in PanIN lesions from Elastase1-CreERT2;KrasG12D and Elastase1-CreERT2;KrasG12D;Notch1lox/lox mice, 3 months following tamoxifen treatment. Scale bar, 50 µm. (B) Expression of Muc-5AC by immunohistochemical staining in PanIN lesions from Elastase1-CreERT2;KrasG12D and Elastase1-CreERT2;KrasG12D;Notch1lox/lox mice following tamoxifen treatment and caerulin-induced pancreatitis. Scale bar, 50 µm. (TIF) [file pone.0052133.s003.tif]
